# Evaluation of BRIP-1 (FANCJ) and FANCI Protein Expression in Ovarian Cancer Tissue

**DOI:** 10.3390/biomedicines12122652

**Published:** 2024-11-21

**Authors:** Mateusz Kozłowski, Dominika Borzyszkowska, Anna Golara, Damian Durys, Katarzyna Piotrowska, Agnieszka Kempińska-Podhorodecka, Aneta Cymbaluk-Płoska

**Affiliations:** 1 Department of Reconstructive Surgery and Gynecological Oncology, Pomeranian Medical University in Szczecin, Al. Powstańców Wielkopolskich 72, 70-111 Szczecin, Polandanka39143@gmail.com (A.G.);; 2 Department of Physiology, Pomeranian Medical University, 70-111 Szczecin, Poland; 3 Department of Medical Biology, Pomeranian Medical University, 70-111 Szczecin, Poland; agnieszka.kempinska.podhorodecka@pum.edu.pl

**Keywords:** ovarian cancer, HGSOC, marker, FANCI, BRIP-1, FANCJ, expression

## Abstract

**Background:** Ovarian cancer is one of the most common cancers in women. Markers associated with ovarian cancer are still being sought. The aim of this study was to evaluate the expression of BRIP-1 (FANCJ) and FANCI proteins in ovarian cancer tissue and to assess these expressions in differentiating the described clinical features. **Methods:** The study enrolled 68 patients with ovarian cancer. The cohort was divided into a HGSOC (high-grade serous ovarian cancer) group and a non-HGSOC group, which represented ovarian cancer other than HGSOC. Immunohistochemical evaluation of FANCI and BRIP-1 (FANCJ) protein expression in ovarian cancer tissue samples was performed. All statistical analyses were performed using StatView software (Carry, NC, USA). **Results:** The FANCI protein mostly showed moderate positive and strong positive expression, while BRIP-1 protein mostly showed no expression or positive expression. Patients with lower expression of FANCI and BRIP-1 showed differences in the clinical stage of HGSOC, which was not observed in patients with higher expression of these proteins. In addition, patients with lower BRIP-1 expression showed differences in menopausal status, which was not observed in patients with higher expression of this protein. **Conclusions:** This study shows that FANCI protein is a marker associated with lower FIGO stage and histologically high-grade cancer in a group of all ovarian cancers and in non-HGSOC.

## 1. Introduction

OC (ovarian cancer) is a huge challenge for modern oncology—according to the WHO, it is the eighth most common cancer in women [1]. More than 324,603 new cases of OC were reported worldwide in 2022 [1]. Large, multicenter randomized trials conducted over the years have failed to show a significant reduction in mortality in patients undergoing screening, which has also led to the conclusion that population-based screening should not be recommended [2,3]. The basis of OC treatment is surgery and chemotherapy. Chemotherapeutic treatment has evolved over the past decades. From adjuvant chemotherapy with platinum/taxanes, then adding new biologic drugs, including bevacizumab—a monoclonal antibody that acts as an angiogenesis inhibitor [4,5]. Another breakthrough was the development of targeted treatment strategies, led by advances in molecular biology [6]. PARP (Poly (adenosine diphosphate-ribose)polymerase) inhibitors are the only approved therapies for genetically determined OC. These inhibitors are highly effective in highly malignant epithelial subtypes harboring the BRCA1/2 pathogenic variant [7,8,9,10].

Descriptions of studies on the expression levels of various proteins such as PD-1 (programmed cell death protein 1), PD-L1 (programmed cell death protein ligand 1), TIM-3 (T-cell immunoglobulin and mucin domain-containing protein-3) or LAG-3 (lymphocyte-activation gene 3) in OC prompts further studies on the expression of these proteins in OC, as well as the search for other new proteins [11,12,13,14]. Among such proteins whose expression is worth studying in ovarian cancer are BRIP1 (BRCA1 interacting protein C-terminal helicase 1) and also FANCI (Fanconi anemia complementation group I).

The BRIP1 protein is part of the DNA damage repair system associated with the BRCA1 protein and owes its full name to this process. This protein is a DNA helicase and is involved in DNA damage repair by breaking down the double helix, and the crucial step is replacing the homologous strand [15,16]. Thus, it plays a role during repair processes, especially in the repair of double-strand breaks using homologous recombination (HR) [17]. The finding of genomic instability in HR-deficient cells and, more significantly, the correlation between cancer propensity and developmental abnormalities and mutations in HR genes highlight the physiological significance of HR. Familial cases of breast and ovarian cancer often involve mutations in the tumor suppressors BRCA1 (breast cancer type 1 susceptibility protein) and BRCA2 (breast cancer type 2 susceptibility protein) [18]. In 13–15% of ovarian cancer cases, germline BRCA1/2 mutations are found. Somatic BRCA1/2 mutations are present in an additional 5–7% of ovarian cancer cases [16]. BRIP1 protein was mostly studied in breast [19,20,21] and ovarian [22,23,24,25] cancer. Missense mutations in BRIP1 may increase both breast and ovarian cancer risk [26]. There are also studies evaluating the association between different BRIP1 variants and cervical cancer risk, a genetic susceptibility to this cancer [27,28]. Mutations in the BRIP1 gene have also been studied for susceptibility to prostate cancer [29].

FANCI is a protein which, in humans, is encoded by the FANCI gene. This protein is involved in the DNA damage repair pathway, specifically the Fanconi anemia (FA) pathway, from which the protein gets its name. FA is a rare autosomal recessive genetic disorder characterized by congenital abnormalities, progressive bone marrow failure and cellular hypersensitivity to DNA cross-linking agents, causing an impaired DNA damage response in certain DNA pathways which may contribute to cancer development [30]. FANCI protein expression has already been studied in various cancers, including hepatocellular carcinoma [31], cutaneous melanoma [32] or prostate cancer, in which FANCI silencing reduces proliferation in p53-expressing prostate cancer cells [33]. The same study also showed that FANCI inactivation can transform cancer cells from a resistant state to one that can be eliminated with DNA-damaging chemotherapy [33]. It was also observed in ovarian cancer, where FANCI silencing promoted DNA damage and sensitized cancer cells to carboplatin [34]. In breast cancer elevated FANCI expression was associated with increased proliferation, while FANCI inhibition has also been shown to sensitize breast cancer cells to the PARP inhibitor in the absence of BRCA mutations [35]. These findings underscore the potential of FANCI targeting to enhance the efficacy of PARP inhibitors in the treatment of not only breast cancer, but also ovarian cancer [33,35]. Each of these studies has demonstrated an important clinical role for the FANCI protein in various cancers [31,32,33,34,35]. Therefore, it seems reasonable to carefully evaluate this protein in ovarian cancer as well.

The aim of this study was to evaluate the expression of BRIP-1 (FANCJ) and FANCI proteins in ovarian cancer tissue and to assess these expressions in differentiating the described clinical features.

## 2. Materials and Methods

### 2.1. Study Design

The study presented here was a retrospective study. Patients with diagnosed and histologically confirmed ovarian cancer were eligible for the study. The entire group was divided into a study group, which was high-grade serous ovarian cancer (HGSOC), and a control group, which was non-high grade serous ovarian cancer (non-HGSOC). The control group consisted of ovarian cancer other than HGSOC. In addition, the group was divided into subgroups taking into account: histological type, grading, clinical stage according to FIGO (International Federation of Gynecology and Obstetrics). The study material was formalin-fixed paraffin-embedded (FFPE) ovarian cancer tissue.

This study was conducted in accordance with the Declaration of Helsinki and approved by the Bioethics Committee of the Pomeranian Medical University in Szczecin (protocol code KB-006/49/2022 on 16 November 2022).

### 2.2. Participation in the Study

FFPE ovarian cancer tissue was used in the retrospective study. The inclusion criterion for the study was the histological diagnosis of ovarian cancer. The criterion for division into the study and control groups was histological type and grading. Exclusion criteria were lack of patient consent, insufficient patient data, treatment history for another malignancy, uncompensated chronic disease, pelvic inflammatory illness, and histological diagnosis of uterine malignancy other than cancer. Finally, 68 patients were qualified for the study. Based on their weight and height, the patients’ body mass indexes (BMIs) were determined at the start of the study. The formula used to determine the BMI was BMI = weight (kg)/height2 (m^2^). The patients were classified into two subgroups based on the results: those with BMI < 25 and those with BMI ≥ 25. Patients were also divided by menopausal status into premenopausal and postmenopausal subgroups.

### 2.3. Immunohistochemical (IHC) Expression of FANCI and BRIP-1 (FANCJ) in Ovarian Cancer Tissue Samples

**Figure 1:**
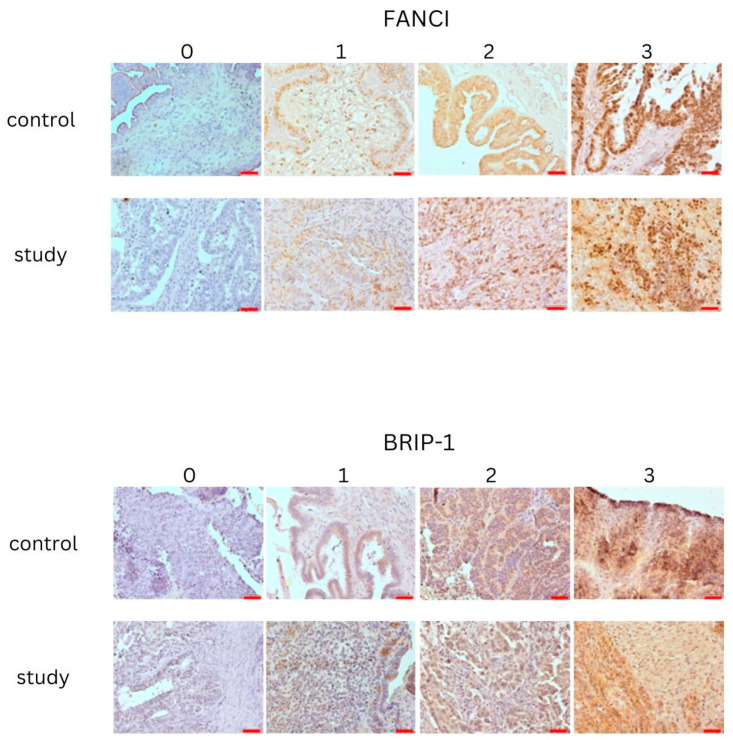
IHC evaluation of FANCI and BRIP: Study and control group samples. 0—negative reaction, 1—positive reaction, 2—moderate positive reaction, 3—strong positive reaction. Objective magnification ×20, scale bars 50 µm.

Heat epitope retrieval was carried out in a microwave oven using citrate buffer pH = 6 (Dako Retrieval Solution, Dako Denmark, Glostrup, Denmark) after sections of ovarian cancer samples (4 μm thick) had been soaked. BLOXALL Endogenous Enzyme Blocking Solution (ImmPRESS^®^ HRP Universal (Horse Anti-Mouse/Rabbit IgG), PLUS Polymer Kit, and Peroxidase, Vector Laboratories, Newark, CA, USA) were used to block the activity of peroxidase after it had cooled to room temperature (RT). It was then rinsed twice with PBS and incubated with 2.5% Normal Horse Serum (ImmPRESS^®^ HRP Universal PLUS Polymer Kit, Peroxidase, Vector Laboratories, Newark, CA, USA). Furthermore, slides were incubated with primary monoclonal antibodies—anti- BRIP-1 (FANCJ) and mouse mononclonal FANCI (Invitrogen, ThermoFisher Scientific, Waltham, MS, USA)—for 1 h in RT. ImmPRESS Universal Antibody Polymer Reagent (ImmPRESS^®^ HRP Universal PLUS Polymer Kit, Peroxidase, Vector Laboratories, USA) was used to incubate the slides after they had been double washed in PBS. The ImmPACT DAB EqV Substrate was used to visualize the reaction following the PBS washing. After visualization, slides were counterstained with hematoxilin (Harris modified Hematoxilin, Sigma, Merck, Darmstadt, Germany) and mounted in Histokitt (CarlRoth, GmbH, Karlsruhe, Germany) mounting medium and evaluated under an Olympus IX81 inverted microscope (Olympus, Germany, Hamburg). Micrographs were collected with CellSens Standard 1.5 software (Olympus, Germany, Hamburg). Positive reaction was described as yellow-to-brown pigmentation found in nuclei and cellular membranes. Intensity of reaction was described as 0—negative reaction, 1—positive reaction, 2—moderate positive reaction, 3—strong positive reaction. Each slide was evaluated by two independent observers. Immunohistochemical expression is illustrated in Figure 1.

### 2.4. Statistical Calculations

All statistical analyses (chi square, odds ratios, confidence intervals) were performed using StatView 5.0 software (Carry, NC, USA). The immunohistochemical expression of FANCI and BRIP-1 in ovarian cancer tissue samples with regards to the analyzed factors was performed using Fisher’s PLSD test. *p* value < 0.05 was considered to be statistically significant. For statistical calculations using Fisher’s PLSD test, the expression intensity of the studied proteins was divided into two groups: the group with expression intensity 0–1 and the group with expression intensity 2–3. Receiver operating characteristic (ROC) curves were used to determine the diagnostic performance, cut-off points, sensitivity, and specificity of FANCI, BRIP-1, and their combination.

### 2.5. Group Characteristics

The total cohort was divided into a study group, which was high-grade serous ovarian cancer (HGSOC), and a control group, which was non-high-grade serous ovarian cancer (non-HGSOC). The control group consisted of ovarian cancer other than HGSOC. Considering age and BMI as continuous characteristics, there was a significant difference in age between the HGSOC and non-HGSOC groups. The difference in BMI between the two groups was not significant. The clinical–demographic characteristics tested were then divided into two subgroups and compared between HGSOC and non-HGSOC. Details of the group characteristics are shown in Table 1.

## 3. Results

### 3.1. Tissue Expression of FANCI Protein

In the group of 0–1 FANCI protein expression intensity, clinical features were compared between HGSOC and non-HGSOC. There was a significant difference in grade (*p* = 0.008), FIGO stage (*p* = 0.04). For menopausal status and BMI, significance was borderline (*p* = 0.05). Differences in histologic type and age were not significant. Details are shown in Table 2.

In the group of 2–3 FANCI protein expression intensity, clinical features were compared between HGSOC and non-HGSOC. There was a significant difference in grade (*p* < 0.0001) and histologic type (*p* < 0.0001). Borderline significance was found for FIGO stage (*p* = 0.07). Differences in menopausal status, BMI, and age were not significant. Details are shown in Table 3.

### 3.2. Tissue Expression of BRIP-1 (FANCJ) Protein

In the group of 0–1 BRIP-1 protein expression intensity, clinical features were compared between HGSOC and non-HGSOC. There was a significant difference in grade (*p* < 0.0001), histologic type (*p* < 0.0001), menopausal status (*p* = 0.04), FIGO stage (*p* = 0.01) Differences in BMI and age were not significant. Details are shown in Table 4.

In the group of 2–3 BRIP-1 protein expression intensity, clinical features were compared between HGSOC and non-HGSOC. There was a significant difference in grade (*p* = 0.005), histologic type (*p* = 0.01). Differences in menopausal status, FIGO stage, BMI and age were not significant. Details are shown in Table 5.

### 3.3. FANCI Protein Expression in the Differentiation of the Described Clinical Features

**Figure 2:**
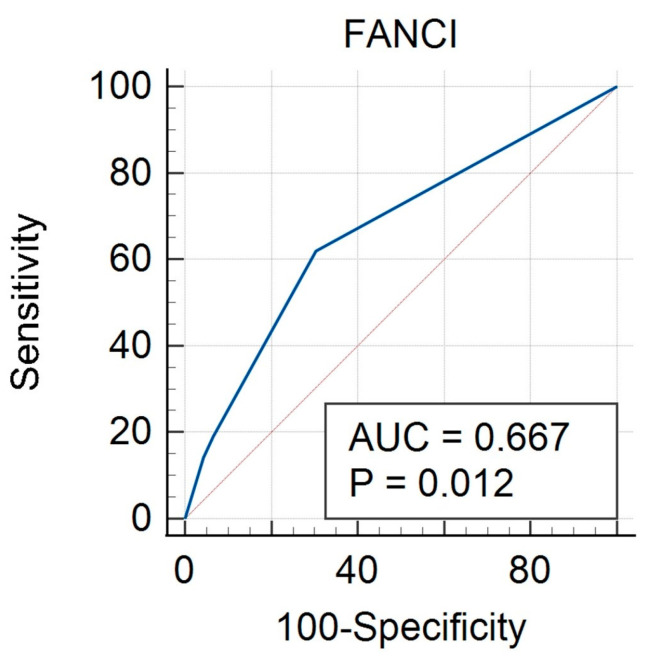
ROC curve for all cancers considering FIGO for FANCI protein.

**Figure 3:**
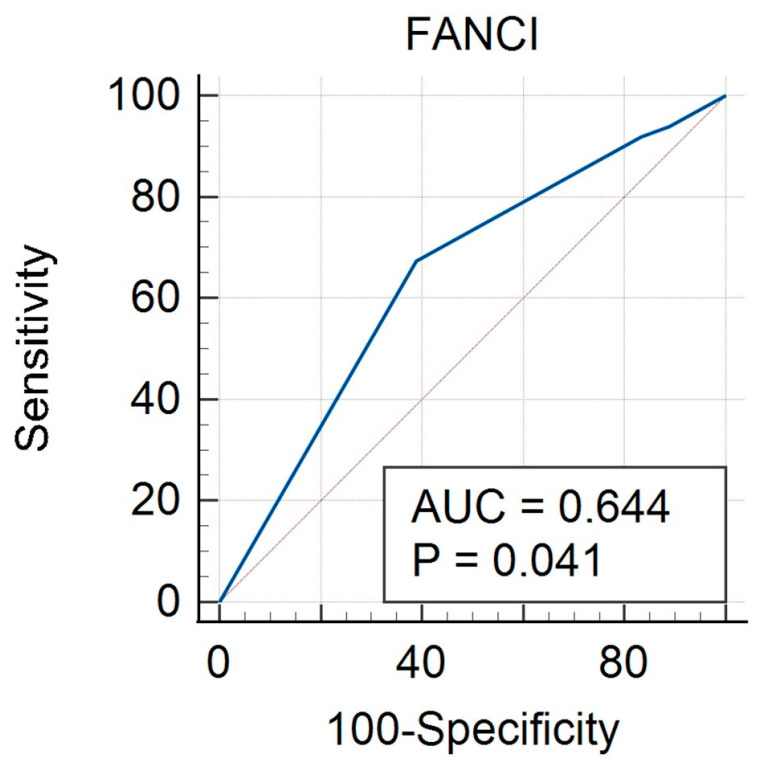
ROC curve for all cancers considering grade for FANCI protein.

**Figure 4:**
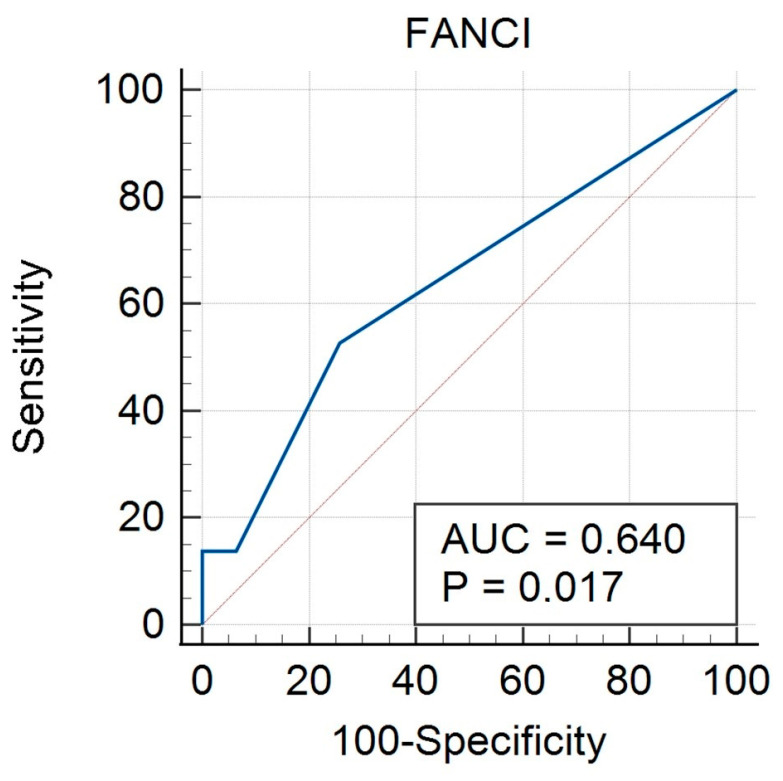
ROC curve for all cancers considering BMI for FANCI protein.

An ROC curve was created for FANCI protein expression taking into account the calculable clinical features studied. The curves were performed both for the group of all cancers (study and control group) and separately for HGSOC and non-HGSOC. In the all-cancer group, there was a significant result for FIGO (AUC 0.667, *p* = 0.012) (Figure 2, Table 6), grade (AUC 0.644, *p* = 0.041) (Figure 3, Table 7) and BMI (AUC 0.640, *p* = 0.017) (Figure 4, Table 8). A non-significant result was obtained for menopausal status (Figure S1), histological type (Figure S2), and age (Figure S3).

The HGSOC group showed non-significant results for FIGO stage (Figure S4), BMI (Figure S5), menopausal status (Figure S6), and age (Figure S7).

**Figure 5:**
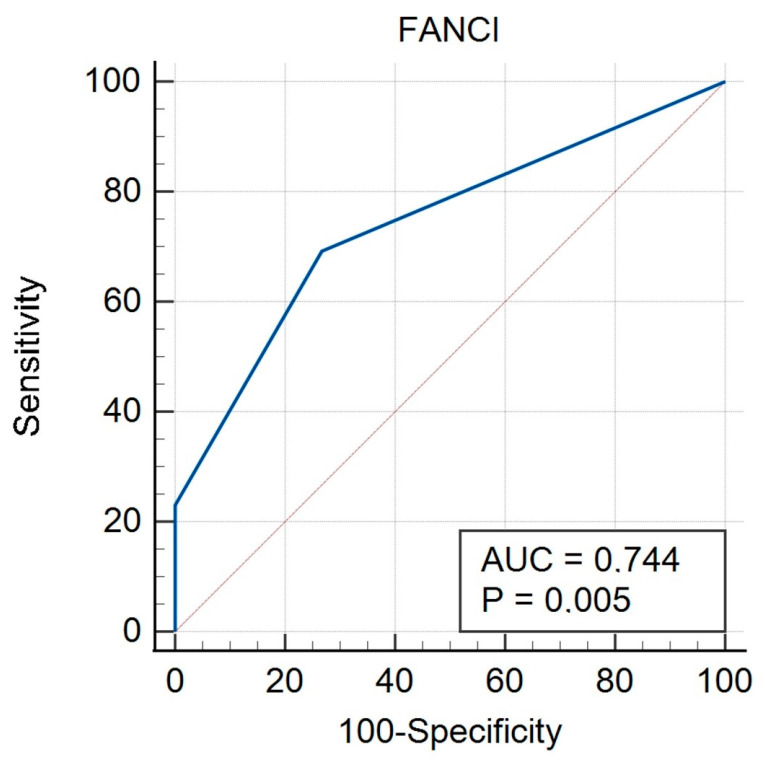
ROC curve for non-HGSOC considering FIGO stage for FANCI protein.

**Figure 6:**
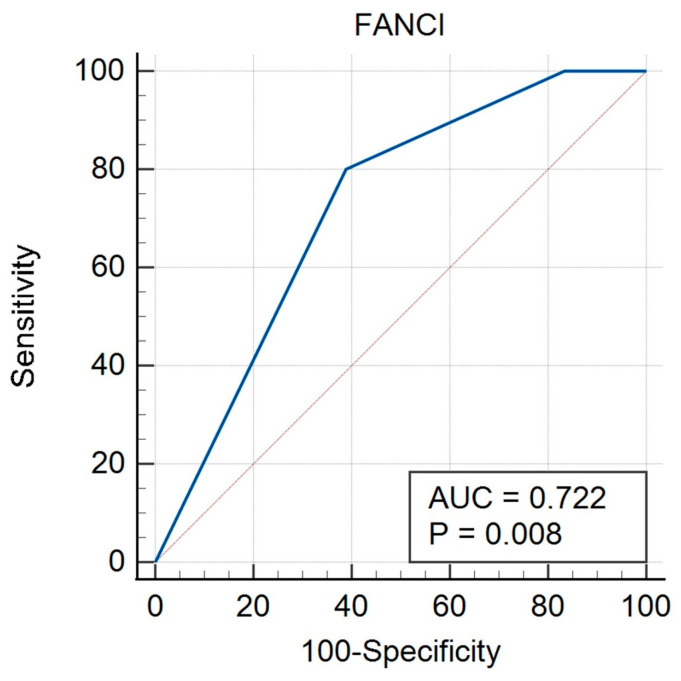
ROC curve for non-HGSOC considering grade for FANCI protein.

**Figure 7:**
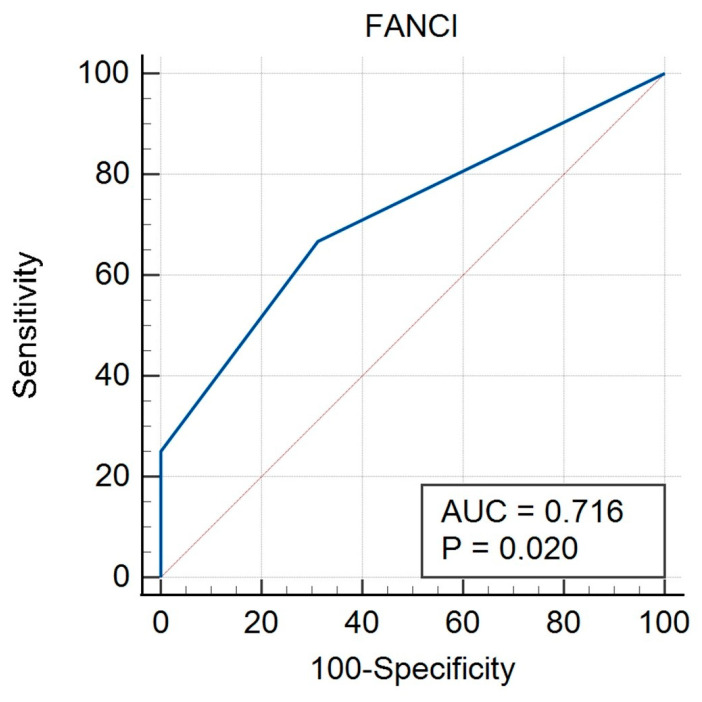
ROC curve for non-HGSOC considering histological type for FANCI protein.

In the non-HGSOC group, there was a significant result for FIGO (AUC 0.774, *p* = 0.005) (Figure 5, Table 9), grade (AUC 0.722, *p* = 0.008) (Figure 6, Table 10), and histological type (AUC 0.716, *p* = 0.020) (Figure 7, Table 11). A non-significant result was obtained for menopausal status (Figure S8), BMI (Figure S9), and age (Figure S10).

### 3.4. BRIP-1 Protein Expression in the Differentiation of the Described Clinical Features

An ROC curve was created for BRIP-1 protein expression taking into account the calculable clinical features studied. The curves were performed both for the group of all cancers (study and control group) and separately for HGSOC and non-HGSOC. In the all-cancer group, there was a non-significant result for BMI (Figure S11), FIGO stage (Figure S12), grade (Figure S13), menopausal status (Figure S14), histological type (Figure S15), and age (Figure S16). In the HGSOC group, there was a non-significant result for BMI (Figure S17), FIGO stage (Figure S18), menopausal status (Figure S19), and age (Figure S20). In the non-HGSOC group, there was a non-significant result for BMI (Figure S21), FIGO stage (Figure S22), grade (Figure S23), menopausal status (Figure S24), histological type (Figure S25), and age (Figure S26).

## 4. Discussion

The “FA pathway”—22 FANC gene products working together in a common DNA repair process—is very important for transposase movement throughout the genome [36,37] and cell resistance to chemotherapy drugs [38]. High-resolution maps enable the development of structurally driven inhibitors or activators of the FA pathway, which may represent chemotherapy-sensitizing agents or even stand-alone single agents in the treatment of cancer and other proliferative disorders.

Using the genetic drift that has been found in the French Canadian (FC) population, Fierheller et al. evaluated the possible pathogenicity of FANCI c.1813C > T; p.L605F by analyzing the allele frequency in FC OC and cancer-free persons. They investigated the functional significance of the encoded p.L605F isoform and its response to medications used to treat OC by in vitro tests and cellulose (HeLa and OC cell lines). Additionally, they looked at FANCI expression in both normal tissues and HGSCs. Among FC patients, familial cases exhibited a higher frequency of c.1813C > T compared to sporadic OC cases (*p* = 0.048). In 2.5 percent of women without cancer, carriers were found. Numerous women who did not develop cancer had host characteristics that could have affected this population’s cancer risk. BRCA1 and BRCA2 pathogenic variant negative OC families had a higher frequency of FANCI c.1813C > T carriers. In 4.1% of Australian OC cases where pathogenic mutations in BRCA1 and BRCA2 were not found, 10 potential FANCI variations, including 10 carriers of FANCI c.1813C > T, were found in non-FC people. In familial OC compared to sporadic OC, candidate variations were substantially more prevalent (*p* = 0.04). In cells expressing the p.L605F isoform, there was a significant reduction in the localization of FANCD2, a component of the FANCI-FANCD2 (ID2) binding complex in the Fanconi anemia (FA) pathway, to sites of induced DNA damage. This isoform exhibited lower expression levels, was destabilized in HeLa and OC cell lines upon treatment with a DNA damaging agent, and was sensitive to cisplatin but not to a poly(ADP-ribose) polymerase inhibitor. Tissue expression of FANCI was evaluated in normal fallopian tubes and in those with high-grade serous carcinoma [39]. Thus, this study differs from ours in its choice of study groups; in our study, we did not include non-cancerous tissues, while we qualified both high-grade and low-grade carcinomas. However, we assessed the intensity of expression similarly—on a four-point scale. Both the Fierheller et al. [39] study and our study showed variable expression. However, in the Fierheller et al. [39] study, it was mostly low-to-moderate staining, while in our study it, was mostly moderate-to-high staining.

Based on genetic research of FANCI c.1813C > T carriers; p.L605F in OC families, FANCI is deemed a new putative gene for ovarian cancer (OC). Fierheller et al. investigated the molecular genetic properties of FANCI. When they examined the FANCI gene in carriers of FANCI c.1813C > T, they discovered that in some cases of high-grade serous ovarian cancer (HGSC), the tumor DNA showed signs of wild-type allele loss. Examining the somatic genetic landscape of OC tumors from FANCI c.1813C > T carriers for copy number alterations, mutational signatures, and mutations in specific genes revealed that the tumor profiles from carriers are similar to characteristics seen in HGSC cases. OC predisposition genes, such as BRCA1 and BRCA2, increase the risk of other cancers, including breast cancer, so researchers also examined the prevalence of FANCI c.1813C > T germline carriers in various cancer types and found overall more carriers among cancer cases compared to tumor-free control (*p* = 0.007). A spectrum of somatic variants in FANCI has also been identified in various cancer types that were not restricted to any specific gene region [40].

In patients with negative-to-low positive intensity of FANCI protein expression, there was a significant difference in the FIGO stage. In the case of menopausal status and BMI, the significance was borderline. However, in patients with moderate-to-high intensity of FANCI protein expression, there was a borderline significance was found for the FIGO stage, although the difference in menopausal status and BMI was not significant. As shown here in several comparisons, statistical significance was borderline. However, given the relatively small group sizes, future studies should be conducted on larger groups, which would perhaps make the results significant. Nevertheless, it is noteworthy that with higher FANCI expression (2 to 3), there was no difference in FIGO stage, whereas differences were observed in lower expressions (0 to 1).

Risk-reducing salpingo-oophorectomy (RRSO) is recommended for women with pathogenic variants (PV) in ovarian cancer risk genes. Personalization of this treatment is based on gene-specific phenotypes, however, the age at diagnosis of ovarian cancer in PV women with moderately penetrant ovarian cancer risk genes is not well studied. Cummings et al. identified in their study that PV in BRIP1 is present in 0.5% of all women studied, but a 3-fold increase is observed in women with a history of ovarian cancer (1.6%) [41]. Germline mutations in BRIP1 are associated with a moderate increase in the risk of epithelial ovarian cancer (EOC), which may have implications for risk prediction and prevention approaches for ovarian cancer. Ramus et al. estimated the relative risk of EOC associated with deleterious mutations in the BRIP1 gene as odds ratios using data from nonfamilial case–control studies. The relative risk (RR) for all EOCs was 11.22, whereas for high-risk EOCs, the relative risk was 14.09. The relative risk has not been proven to change with age (*p* = 0.55). The estimated cumulative risk at age 80 was 5.8%. The association of ovarian cancer with BRIP1 variants was RR = 3.94, 95% CI = 2.44 to 6.22, *p* = 1 × 10^−8^) [22]. As shown above, BRIP-1 has so far been studied for mutations in this gene and their association with ovarian cancer. To date, there is a lack of studies evaluating tissue expression of BRIP-1 protein, especially considering the HGSOC group. When comparing tissue expression of BRIP-1, it is important to note menopausal status and FIGO stage. We observed that in the group with lower expression (0 to 1), there were significant differences between HGSOC and non-HGSOC in menopausal status and FIGO stage. In contrast, in the group with higher expression (2 to 3), these differences were not significant. We wanted to compare the proteins we studied to other articles that described known HGSOC biomarkers. About 15–20% patients with HGSOC have BRCA1/2 mutations [42]. Lheureux et al. detailed other genes with moderate penetrance, including the BRIP1 gene, which, although its mutation frequency has individually been described as rare, genes with moderate penetrance may together be responsible for about 5% of EOC. Therefore, in women with HGSOC, genetic testing includes both BRCA1/2 and other susceptibility genes, including BRIP1 [42]. Jones et al. describes that BRCA1/2 mutations in the germline are most often associated with the development of the HGSOC histotype. In the same study, they detailed BRIP1 as one of the likely susceptibility genes, particularly for the HGSOC histotype [43]. A significant percentage of HGSOC cases are also due to non-BRCA mutations that may be associated with a poor prognosis. For example, a paper by Yao Q et al. detailed that patients with HGSOC, for example, had 20.5% mutations in the BRCA1 gene, 7.3% mutations in BRCA2, 1.8% in the BRIP1 gene, and 0.7% each in the CHEK2 and FANCI genes, respectively [44].

A limitation of this study was the number of patients. The entire cohort consisted of 68 cases, which—when divided—resulted in even smaller subgroups. Increasing the group of examined patients could change some of the results to statistically significant ones. As shown in the literature, mainly polymorphisms of FANCI and BRIP-1 genes and their association with ovarian cancer have been studied. Nevertheless, there are few studies on FANCI AND BRIP-1, especially studies evaluating tissue expression of these proteins. This represents both an advantage and a disadvantage for our study. On the one hand, there are few data in the literature with which to compare the results of our study. On the other hand, the study we present is one of the first to present immunohistochemical evaluation of tissue expression of FANCI and BRIP-1 in ovarian cancer tissue. Both the literature data and the results presented in this article indicate that FANCI and BRIP-1 proteins are associated with ovarian cancer. At the same time, the limitations of this study indicate the prospect of studies to be conducted in the future. Certainly, a similar study to the initial study presented here should be conducted, but with a larger number of patients. It would also be appropriate to focus on the relationship of selected polymorphisms of the proteins studied to their tissue expression and to clinico-demographic characteristics.

## 5. Conclusions

The FANCI protein mostly showed moderately positive and strong positive expression, while BRIP-1 protein mostly showed no expression or positive expression. Patients with lower expressions of FANCI and BRIP-1 showed differences in the clinical stage of HGSOC, which was not observed in patients with higher expression of these proteins. In addition, patients with lower BRIP-1 expression showed differences in menopausal status, which was not observed in patients with higher expression of this protein. This study shows that FANCI protein is a marker associated with lower FIGO stage and histologically high-grade cancer in a group of all ovarian cancers and in non-HGSOC.

## Figures and Tables

**Table 1:** Clinical–demographic characteristics.

Parameters	Total Cohort (*n* = 68)	Non-HGSOC (*n* = 28)	HGSOC (*n* = 40)	*p*-Value *	OR (95% CI)
Median (IQR)					
Age (years old)	61.0 (20)	57.0 (19)	64.5 (15)	0.03	
BMI (kg/m^2^)	25.1 (6.5)	25.1 (6.9)	25.4 (6.3)	NS	
Number (%)					
FANCI protein expression intensity					0.9 (0.2–5.2)
0–1	7 (10.3%)	3 (10.7%)	4 (11.1%)		0.5 (0.2–1.5)
2–3	61 (89.7%)	25 (89.3%)	36 (88.9%)		
BRIP-1 protein expression intensity					
0–1	49 (72.1%)	18 (64.3%)	31 (77.5%)		
2–3	19 (27.9%)	10 (35.7%)	9 (22.5%)		
Age (years old)					
<65	39 (57.3%)	19 (67.9%)	20 (50%)	NS	
≥65	29 (42.6%)	9 (32.1%)	20 (50%)		
BMI					
<25	31 (45.6%)	13 (46.4%)	18 (45%)	NS	
≥25	37 (54.4%)	15 (53.6%)	22 (55%)		
Menopausal status					
Premenopausal	22 (32.4%)	12 (42.9%)	10 (25%)	NS	
Postmenopausal	46 (67.6%)	16 (57.1%)	30 (75%)		
Grade					
High (G2 + G3)	50 (73.5%)	10 (35.7%)	40 (100%)	0.0001	
Low (G1)	18 (26.5%)	18 (64.3%)	0 (0%)		
Histological type					
Serous	52 (76.5%)	12 (42.8%)	40 (100%)	0.0001	
Non-serous	16 (23.5%)	16 (57.2%)	0 (0%)		
FIGO stage					
I–II	21 (31.1%)	13 (46.4%)	8 (20%)	0.02	
III–IV	47 (69.1%)	15 (53.6%)	32 (80%)		

* Fisher’s exact *p*-value, OR—odds ratio, CI—confidence interval, NS—non-significant.

**Table 2:** Comparison of clinical characteristics in group 0–1 of FANCI protein expression intensity between HGSOC and non-HGSOC.

	Non-HGSOC (*n* = 3)	HGSOC (*n* = 4)	*p*-Value *
Group 0–1 of FANCI protein expression intensity			
Grade			
High	0 (0%)	4 (100%)	0.008
Low	3 (100%)	0 (0%)	
Histological type Serous Non-serous	3 (100%) 0 (0%)	4 (100%) 0 (0%)	NS
Menopausal status			
Premenopausal	2 (66.7%)	0 (0%)	0.05
Postmenopausal	1 (33.3%)	4 (100%)	
FIGO stage			
I–II	3 (100%)	1 (25%)	0.04
III–IV	0 (0%)	3 (75%)	
BMI			
<25	1 (33.3%)	1 (25%)	0.05
>25	2 (66.7%)	3 (75%)	
Age			
<65 years old	2 (66.7%)	2 (50%)	NS
>65 years old	1 (33.3%)	2 (50%)	

* Fisher’s exact *p*-value, NS—non-significant.

**Table 3:** Comparison of clinical characteristics in group 2–3 of FANCI protein expression intensity between HGSOC and non-HGSOC.

	Non-HGSOC (*n* = 25)	HGSOC (*n* = 36)	*p*-Value *
Group of 2–3 FANCI protein expression intensity			
Grade			
High	10 (40%)	36 (100%)	<0.0001
Low	15 (60%)	0 (0%)	
Histological type			
Serous	9 (36%)	36 (100%)	<0.0001
Non-serous	16 (64%)	0 (0%)	
Menopausal status			
Premenopausal	10 (40%)	10 (27.8%)	NS
Postmenopausal	15 (60%)	26 (72.2%)	
FIGO stage			
I–II	10 (40%)	7 (19.4%)	0.07
III–IV	15 (60%)	29 (80.6%)	
BMI			
<25	12 (48%)	17 (47.2%)	NS
>25	13 (52%)	19 (52.8%)	
Age			
<65 years old	17 (68%)	18 (50%)	NS
>65 years old	8 (32%)	18 (50%)	

* Fisher’s exact *p*-value, NS—non-significant.

**Table 4:** Comparison of clinical characteristics in group 0–1 of BRIP-1 protein expression intensity between HGSOC and non-HGSOC.

	Non-HGSOC (*n* = 18)	HGSOC (*n* = 31)	*p*-Value *
Group of 0–1 BRIP-1 protein expression intensity			
Grade			
High	6 (33.3%)	31 (100%)	<0.0001
Low	12 (66.7%)	0 (0%)	
Histological type			
Serous	7 (38.9%)	31 (100%)	<0.0001
Non-serous	11 (61.1%)	0 (0%)	
Menopausal status			
Premenopausal	9 (50%)	7 (22.6%)	0.04
Postmenopausal	9 (50%)	24 (77.4%)	
FIGO stage			
I–II	9 (50%)	5 (16.1%)	0.01
III–IV	9 (50%)	26 (83.9%)	
BMI			
<25	7 (38.9%)	13 (41.9%)	NS
>25	11 (61.1%)	18 (58.1%)	
Age			
<65 years old	14 (77.8%)	17 (54.8%)	NS
>65 years old	4 (22.2%)	14 (45.2%)	

* Fisher’s exact *p*-value, NS—non-significant.

**Table 5:** Comparison of clinical characteristics in the group of 2–3 BRIP-1 protein expression intensity between HGSOC and non-HGSOC.

	Non-HGSOC (*n* = 10)	HGSOC (*n* = 9)	*p*-Value *
Group of 2–3 BRIP-1 protein expression intensity			
Grade			
High	4 (40%)	9 (100%)	0.005
Low	6 (60%)	0 (0%)	
Histological type			
Serous	5 (50%)	9 (100%)	0.01
Non-serous	5 (50%)	0 (0%)	
Menopausal status			
Premenopausal	3 (30%)	3 (33.3%)	NS
Postmenopausal	7 (70%)	6 (66.7%)	
FIGO stage			
I–II	4 (40%)	3 (33.3%)	NS
III–IV	6 (60%)	6 (66.7%)	
BMI			
<25	6 (60%)	5 (55.6%)	NS
>25	4 (40%)	4 (44.4%)	
Age			
<65 years old	5 (50%)	3 (33.3%)	NS
>65 years old	5 (50%)	6 (66.7%)	

* Fisher’s exact *p*-value, NS—non-significant.

**Table 6:** Criterion values and coordinates of the ROC curve for all cancers considering FIGO for FANCI protein.

Criterion	Sensitivity	95% CI	Specificity	95% CI	+LR	−LR
<0	0.00	0.0–16.1	100.00	92.3–100.0		1.00
≤0	14.29	3.0–36.3	95.65	85.2–99.5	3.29	0.90
≤1	19.05	5.4–41.9	93.48	82.1–98.6	2.92	0.87
≤2	61.90	38.4–81.9	69.57	54.2–82.3	2.03	0.55
≤3	100.00	83.9–100.0	0.00	0.0–7.7	1.00	

**Table 7:** Criterion values and coordinates of the ROC curve for all cancers considering grade for FANCI protein.

Criterion	Sensitivity	95% CI	Specificity	95% CI	+LR	−LR
≥0	100.00	92.7–100.0	0.00	0.0–18.5	1.00	
>0	93.88	83.1–98.7	11.11	1.4–34.7	1.06	0.55
>1	91.84	80.4–97.7	16.67	3.6–41.4	1.10	0.49
>2	67.35	52.5–80.1	61.11	35.7–82.7	1.73	0.53
>3	0.00	0.0–7.3	100.00	81.5–100.0		1.00

**Table 8:** Criterion values and coordinates of the ROC curve for all cancers considering BMI for FANCI protein.

Criterion	Sensitivity	95% CI	Specificity	95% CI	+LR	−LR
<0	0.00	0.0–9.7	100.00	88.8–100.0		1.00
≤0	13.89	4.7–29.5	100.00	88.8–100.0		0.86
≤1	13.89	4.7–29.5	93.55	78.6–99.2	2.15	0.92
≤2	52.78	35.5–69.6	74.19	55.4–88.1	2.05	0.64
≤3	100.00	90.3–100.0	0.00	0.0–11.2	1.00	

**Table 9:** Criterion values and coordinates of the ROC curve for non-HGSOC considering FIGO stage for FANCI protein.

Criterion	Sensitivity	95% CI	Specificity	95% CI	+LR	−LR
<0	0.00	0.0–24.7	100.00	78.2–100.0		1.00
≤1	23.08	5.0–53.8	100.00	78.2–100.0		0.77
≤2	69.23	38.6–90.9	73.33	44.9–92.2	2.60	0.42
≤3	100.00	75.3–100.0	0.00	0.0–21.8	1.00	

**Table 10:** Criterion values and coordinates of the ROC curve for non-HGSOC considering grade for FANCI protein.

Criterion	Sensitivity	95% CI	Specificity	95% CI	+LR	−LR
≥0	100.00	69.2–100.0	0.00	0.0–18.5	1.00	
>1	100.00	69.2–100.0	16.67	3.6–41.4	1.20	0.00
>2	80.00	44.4–97.5	61.11	35.7–82.7	2.06	0.33
>3	0.00	0.0–30.8	100.00	81.5–100.0		1.00

**Table 11:** Criterion values and coordinates of the ROC curve for non-HGSOC considering histological type for FANCI protein.

Criterion	Sensitivity	95% CI	Specificity	95% CI	+LR	−LR
<0	0.00	0.0–26.5	100.00	79.4–100.0		1.00
≤1	25.00	5.5–57.2	100.00	79.4–100.0		0.75
≤2	66.67	34.9–90.1	68.75	41.3–89.0	2.13	0.48
≤3	100.00	73.5–100.0	0.00	0.0–20.6	1.00	

## Data Availability

The data presented in this study are available from the corresponding author, M.K., upon reasonable request.

## Supplementary Materials

The following supporting information can be downloaded at: https://www.mdpi.com/article/10.3390/biomedicines12122652/s1, Figure S1: ROC curve for all cancers considering menopausal status for FANCI protein; Figure S2: ROC curve for all cancers considering histological type for FANCI protein; Figure S3: ROC curve for all cancers considering age for FANCI protein; Figure S4: ROC curve for HGSOC considering FIGO stage for FANCI protein; Figure S5: ROC curve for HGSOC considering BMI for FANCI protein; Figure S6: ROC curve for HGSOC considering menopausal status for FANCI protein; Figure S7: ROC curve for HGSOC considering age for FANCI protein; Figure S8: ROC curve for non-HGSOC considering menopausal status for FANCI protein; Figure S9: ROC curve for non-HGSOC considering BMI for FANCI protein; Figure S10: ROC curve for non-HGSOC considering age for FANCI protein; Figure S11: ROC curve for all cancers considering BMI for BRIP-1 protein; Figure S12: ROC curve for all cancers considering FIGO stage for BRIP-1 protein; Figure S13: ROC curve for all cancers considering grade for BRIP-1 protein; Figure S14: ROC curve for all cancers considering menopausal status for BRIP-1 protein; Figure S15: ROC curve for all cancers considering histological type for BRIP-1 protein; Figure S16: ROC curve for all cancers considering age for BRIP-1 protein; Figure S17: ROC curve for HGSOC considering BMI for BRIP-1 protein; Figure S18: ROC curve for HGSOC considering FIGO stage for BRIP-1 protein; Figure S19: ROC curve for HGSOC considering menopausal status for BRIP-1 protein; Figure S20: ROC curve for HGSOC considering age for BRIP-1 protein; Figure S21: ROC curve for non-HGSOC considering BMI for BRIP-1 protein; Figure S22: ROC curve for non-HGSOC considering FIGO stage for BRIP-1 protein; Figure S23: ROC curve for non-HGSOC considering grade for BRIP-1 protein; Figure S24: ROC curve for non-HGSOC considering menopausal status for BRIP-1 protein; Figure S25: ROC curve for non-HGSOC considering histological type for BRIP-1 protein; Figure S26: ROC curve for non-HGSOC considering age for BRIP-1 protein.
