# Supplementary material for: Evaluation of BRIP-1 (FANCJ) and FANCI Protein Expression in Ovarian Cancer Tissue
Source: Biomedicines. 2024 Nov 21;12(12):2652. doi: 10.3390/biomedicines12122652 (PMC11673538; doi:10.3390/biomedicines12122652)
Supplement: Supplementary file 1 [file biomedicines-12-02652-s001.zip › biomedicines-3304856-supplementary.pdf]

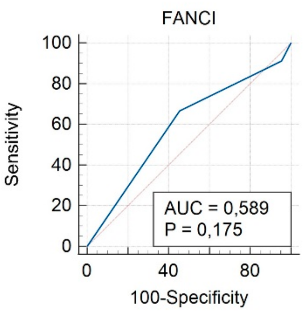

Figure S1. ROC curve for all cancers considering menopausal status for FANCI protein

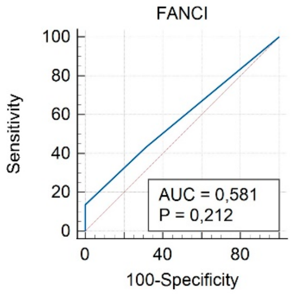

Figure S2. ROC curve for all cancers considering histological type for FANCI protein

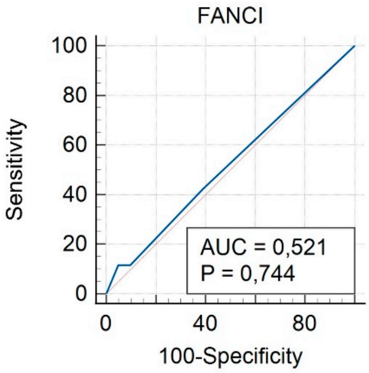

Figure S3. ROC curve for all cancers considering age for FANCI protein

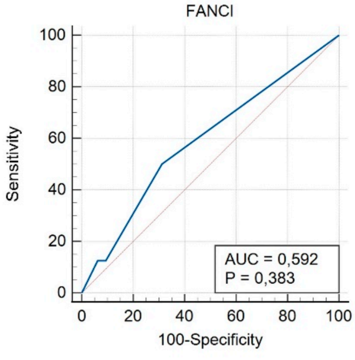

Figure S4. ROC curve for HGSOC considering FIGO stage for FANCI protein

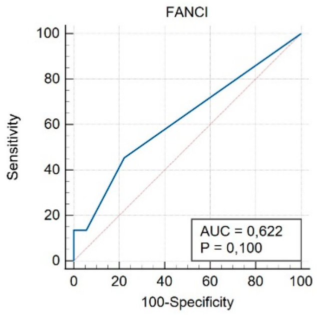

Figure S5. ROC curve for HGSOC considering BMI for FANCI protein

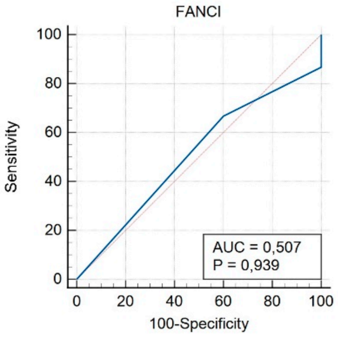

Figure S6. ROC curve for HGSOC considering menopausal status for FANCI protein

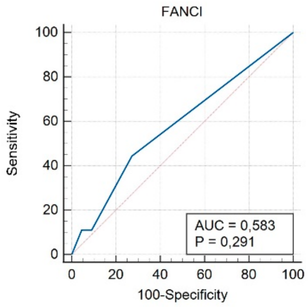

Figure S7. ROC curve for HGSOC considering age for FANCI protein

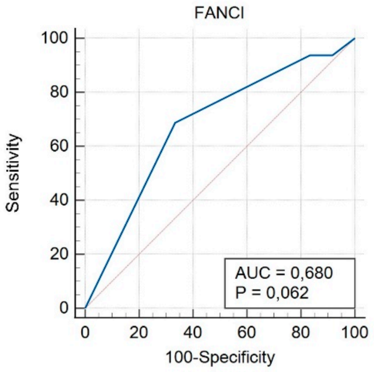

Figure S8. ROC curve for non-HGSOC considering menopausal status for FANCI protein

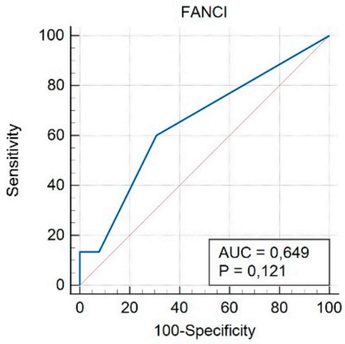

Figure S9. ROC curve for non-HGSOC considering BMI for FANCI protein

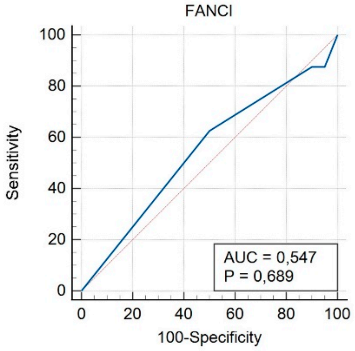

Figure S10. ROC curve for non-HGSOC considering age for FANCI protein

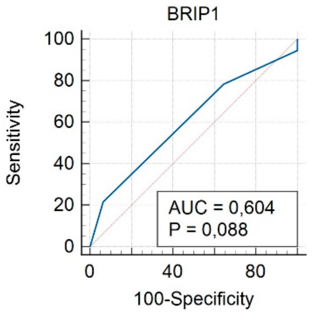

Figure S11. ROC curve for all cancers considering BMI for BRIP-1 protein

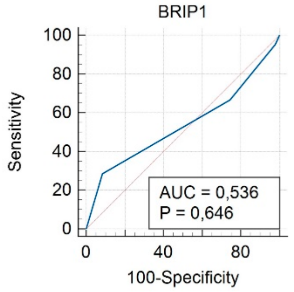

Figure S12. ROC curve for all cancers considering FIGO stage for BRIP-1 protein

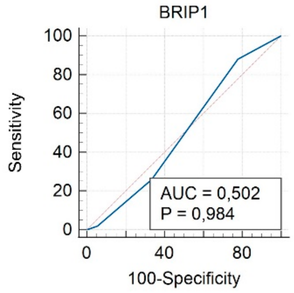

Figure S13. ROC curve for all cancers considering grade for BRIP-1 protein

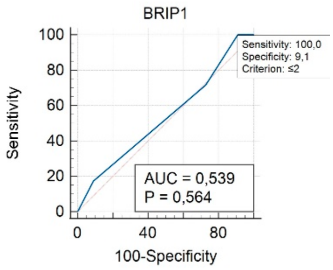

Figure S14. ROC curve for all cancers considering menopausal status for BRIP-1 protein

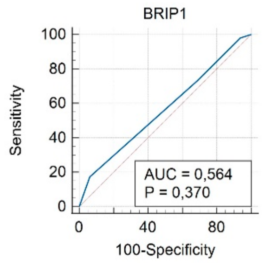

Figure S15. ROC curve for all cancers considering histological type for BRIP-1 protein

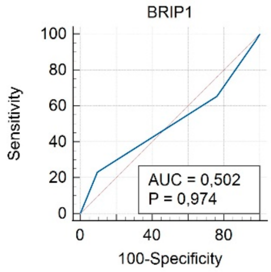

Figure S16. ROC curve for all cancers considering age for BRIP-1 protein

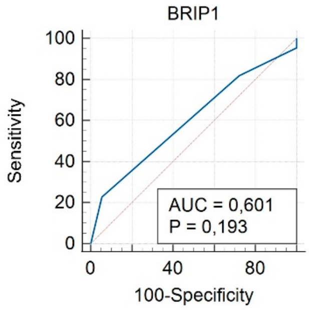

Figure S17. ROC curve for HGSOC considering BMI for BRIP-1 protein

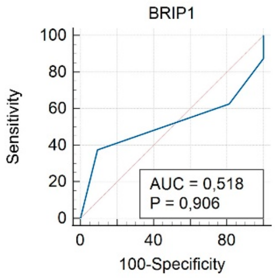

Figure S18. ROC curve for HGSOC considering FIGO stage for BRIP-1 protein

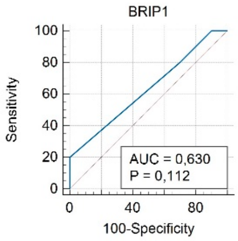

Figure S19. ROC curve for HGSOC considering menopausal status for BRIP-1 protein

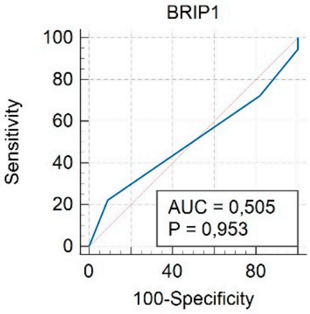

Figure S20. ROC curve for HGSOC considering age for BRIP-1 protein

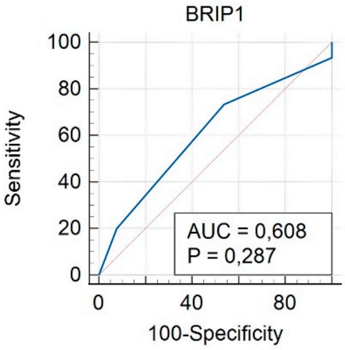

Figure S21. ROC curve for non-HGSOC considering BMI for BRIP-1 protein

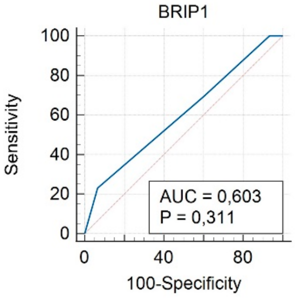

Figure S22. ROC curve for non-HGSOC considering FIGO stage for BRIP-1 protein

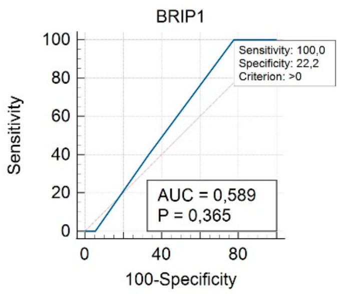

Figure S23. ROC curve for non-HGSOC considering grade for BRIP-1 protein

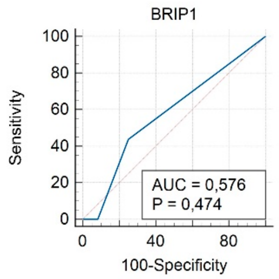

Figure S24. ROC curve for non-HGSOC considering menopausal status for BRIP-1 protein

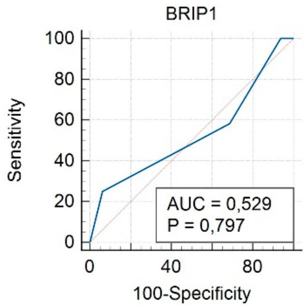

Figure S25. ROC curve for non-HGSOC considering histological type for BRIP-1 protein

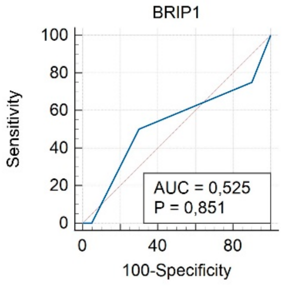

Figure S26. ROC curve for non-HGSOC considering age for BRIP-1 protein
